# Dysregulation of macrophage development and phenotype in diabetic human macrophages can be rescued by Hoxa3 protein transduction

**DOI:** 10.1371/journal.pone.0223980

**Published:** 2019-10-18

**Authors:** Salma Alrdahe, Hadeel Al Sadoun, Tanja Torbica, Edward A. McKenzie, Frank L. Bowling, Andrew J. M. Boulton, Kimberly A. Mace

**Affiliations:** 1 Division of Cell Matrix Biology & Regenerative Medicine, Faculty of Biology, Medicine and Health, Manchester Academic Health Science Centre, University of Manchester, Manchester, United Kingdom; 2 Stem Cell Unit, King Fahad Medical Research Center, Department of Laboratory Medical Technology, Faculty of Applied Medical Sciences, King Abdulaziz University, Saudi Arabia; 3 Manchester Institute of Biotechnology, University of Manchester, Manchester, United Kingdom; 4 Division of Diabetes, Endocrinology & Gastroenterology, Faculty of Biology, Medicine and Health, Manchester Academic Health Science Centre, University of Manchester, Manchester, United Kingdom; Albert Einstein College of Medicine, UNITED STATES

## Abstract

Controlled inflammatory responses of myeloid cells recruited to wounds are essential for effective repair. In diabetes, the inflammatory response is prolonged and augmented over time, with increased myeloid cells present in the wound that fail to switch from a pro-inflammatory phenotype to a pro-healing phenotype. These defects lead to delayed angiogenesis and tissue repair and regeneration, and contribute to chronic wound formation. In mouse models of diabetes, this aberrant phenotype is partially mediated by stable intrinsic changes to the developing myeloid cells in the bone marrow, affecting their maturation and polarization potential. Previous studies have shown that freshly isolated peripheral blood mononuclear cells from diabetic patients are more inflammatory than non-diabetic counterparts. However, the phenotype of macrophages from human diabetic patients has not been well characterized. Here we show that diabetic-derived human macrophages cultured for 6 days in vitro maintain a pro-inflammatory priming and hyperpolarize to a pro-inflammatory phenotype when stimulated with LPS and INF-ɣ or TNF. In addition, diabetic-derived macrophages show maturation defects associated with reduced expression of the *RUNX1* transcription factor that promotes myeloid cell development. Targeting intrinsic defects in myeloid cells by protein transduction of the Hoxa3 transcription factor can rescue some inflammation and maturation defects in human macrophages from diabetic patients via upregulation of *Runx1*. In addition, Hoxa3 can modulate the levels of p65/NF-κB and histone acetyltransferase and deacetylase activity, as well as inhibit acetylation of the *TNF* promoter. Altogether, these results show a link between myeloid cell maturation and inflammatory responses, and that diabetes induces intrinsic changes to human myeloid cells that are maintained over time, as well as potentially therapeutic Hoxa3-mediated mechanisms of controlling the inflammatory response in diabetes.

## Introduction

Inflammation is a fundamental biological process that not only protects the host against infection from pathogens but also drives the repair and healing response following injury. Failure to appropriately regulate inflammation results in a plethora of complications, including non-healing wounds that can occur in patients with diabetes mellitus. The cost of wound care in the United States is estimated between 28.1–96.8 billion dollars annually, and complications from wounds affect around 8.2 million people on Medicare each year [1]. Diabetic foot ulcers and their complications are estimated to cost the Medicare system 11.7 billion dollars annually, making up approximately 12–42% of the total amount. Although a proportion of these cases are manageable, some are incurable and represent the main cause of lower limb amputation in the developed world [2,3].

Although, the etiology of chronic wounds is not well understood, chronic inflammation is associated with their development [3], therefore there is a critical need to better understand the causes of chronic inflammation in the wound environment and identify potential preventative or curative therapies. In the past decade, several studies have focused on the role of myeloid cells in the normal healing response and how they can be dramatically dysregulated in diabetes, with a particular focus on macrophages, due to their plasticity and array of polarization phenotypes [4–8]. In the early stage of wound healing, M1-like pro-inflammatory macrophages are predominant. These cells destroy and remove damaged or dead cells and bacteria to sterilize the wound area. As inflammation proceeds, macrophages remove apoptosed neutrophils in the process of efferocytosis. This process can result in a phenotypic switch from an M1-like macrophage to an M2-like anti-inflammatory state that promotes resolution of inflammation [8–10].

As healing proceeds to the next phase of tissue proliferation and regeneration, macrophages adopt a pro-healing phenotype, secreting growth factors that promote tissue regeneration, neovascularization and wound closure, including vascular endothelial growth factor (VEGF) and transforming growth factor beta (TGFβ) [5,8,10,11]. Mouse models of diabetes have demonstrated that in diabetic wounds, macrophages are dysregulated and unable to resolve inflammation, remaining in a prolonged inflammatory phenotype without switching to the reparative fate, resulting in severely impaired wound healing [12–15]. These studies also suggest the aberrant diabetic macrophage phenotype begins early in their development in the bone marrow and link defects in polarization phenotype to defects in maturation. Diabetic mouse monocytes, macrophages and granulocytes express lower levels of maturation markers such as CD11b, CCR2, F4/80 and CxCR2 due to lower levels of myeloid cell differentiation regulators, such as CCAAT enhancer binding protein alpha (CEPBα) and SPI1, as well as deregulation of chromatin remodelling enzymes [12,16]. However, it remained unclear whether the aberrant differentiation observed in diabetic mouse macrophages was also found in human diabetic patients. Imbalances in M1/M2 phenotypes have been reported in the human obese population, together with increased M1 cytokine levels, which have been shown to promote adipose tissue dysfunction and insulin resistance even before the occurrence of diabetes, and which can be used as a predictor for type II diabetes in obese populations [reviewed in [17]].

Because macrophage dysregulation in diabetes likely arises early in myeloid cell development, targeting cell-intrinsic factors that cause dysregulation in maturation and polarization may be a more effective strategy than solely dampening the excessive inflammatory signals they receive in the wound. Hoxa3 is an anti-inflammatory transcription factor that is expressed in acute wounds but significantly reduced in diabetic wounds [18]. We previously showed that overexpression of Hoxa3 in murine macrophages successfully inhibited the inflammatory phenotype in classically activated bone marrow-derived macrophages and suppressed excessive production of inflammatory cytokines such as IL-6, TNF and nitric oxide. In addition, sustained expression of Hoxa3 promoted maturation in macrophages via upregulation of the transcription factor *Spi1/Pu*.*1* [19]. However, until now, the effect of Hoxa3 on the monocyte/macrophage population derived from human diabetic patients has not been investigated. In this study, we hypothesized that peripheral blood monocyte-derived macrophages from diabetic patients would show similar defects in maturation and phenotype to those seen in diabetic mice. We also hypothesized that Hoxa3 protein-based treatment would rescue these defects in human macrophages. Here we show that some of the Hoxa3 effects observed in mouse macrophages can be translated to human diabetic macrophages and that Hoxa3 protein inhibits the chronic inflammatory state, possibly through promoting maturation via upregulation of myeloid cell transcription factors, modulating NF-κB activation, and modulating chromatin modifying enzyme activity. Thus, Hoxa3 may have important benefits as a potential therapeutic target in future.

## Materials and methods

### Collection of human blood samples, culture of peripheral blood monocytes, and differentiation of macrophages

All procedures involving human peripheral blood were approved by the Northwest Research Ethics Committee (reference 16/YH/0415), NHS HRA, and NHS R&D (IRAS project ID 188513). Informed consent was obtained from all participants prior to blood collection.

Inclusion criteria were as follows: males or females (pre-menopausal) attending Central Manchester University Hospitals NHS Foundation Trust (CMFT) diabetes clinics, recruited via local media (e.g., hospital newsletter) or on the Help Beat Diabetes database, or healthy volunteers recruited through a University of Manchester electronic newsletter or word of mouth. Participants had to be 18–65 years old, and if diabetic, had to have been diagnosed with diabetes at least 1 year prior to this study. Patients were diagnosed with diabetes if they had satisfied the following criteria:

- Current treatment with oral hypoglycaemic or non-insulin injectable therapies or
- If on insulin, this was started at least two years after the original diagnosis of diabetes

Exclusion criteria included diabetic patients or healthy volunteers with secondary health complications such as foot ulcers, severe renal impairment, significant peripheral vascular disease (with or without prior clinical interventions), autoimmune conditions or other inflammatory diseases, thyroid disease or malignant disease. Patients or volunteers receiving anti-mitotic therapeutic agents or steroids (or with a history of steroid therapy), those with anemia (defined as haemoglobin levels <110 g/L), a history of transplant, or those who did not consent, were also excluded. In addition, for healthy volunteers, no known current or history of diabetes was required to participate. Both males and females were included in the study and age and sex-matched as closely as possible (see Table 1).

**Table 1:** Summary of data for patients and healthy volunteers used in this study.

Sample type	No. females	No. males	Mean age
Healthy	12	5	39
Diabetic	4	10	54

Blood samples were collected into EDTA vacutainer tubes (Becton Dickson, 367525) and diluted 1:1 in RPMI-1640 medium (RPMI; Sigma Aldrich, R8758). Peripheral blood mononuclear cells (PBMCs) were isolated by density gradient centrifugation using Histopaque (Sigma Aldrich, 10771) following the manufacturer’s instructions.

For macrophage differentiation, monocytes were isolated from PBMCs by negative selection using EasySep Human Monocyte Enrichment Kit without CD16 depletion (StemCell Technologies, 19058) following the manufacturer’s protocol. Isolated monocytes were cultured in RPMI medium supplemented with 1% penicillin/streptomycin (P/S) (Sigma Aldrich, P0781), 10% fetal bovine serum (FBS) (Sigma Aldrich, F9665) and 50 ng/ml recombinant human macrophage colony stimulating factor (M-CSF) (PeproTech, 300–25). On day 3 post culture, cells were fed with one volume of the supplemented RPMI medium and by day 6, cells were differentiated into macrophages and ready for further treatment. Between 3 and 8 samples were used for each group, as indicated in figure legends.

### Morphological analyses, viability assays and colony forming unit assays

Isolated PBMCs were purified using CD11b-labeled magnetic beads (StemCell Technologies, EasySep Human APC Positive Selection Kit, 18451), according to the manufacturer’s protocol. CD11b^+^ PBMCs were cultured in Hoxa3-mCherry- or mCherry-conditioned medium for 48 hours (morphological analyses), 8 days (viability assays), or 15 days (colony forming unit [CFU] assays). Morphological assays were performed as follows: 1x10^5^ fresh or cultured CD11b^+^ PBMCs were resuspended in 200 μL isolation buffer (3% FBS in PBS) and spun onto slides for 5 minutes at 800rpm using a Cytospin III (Shandon). Slides were dried overnight, then fixed in methanol for 5 minutes before staining for 45 minutes in 5% fresh Giemsa solution. Dry slides were mounted with Neomount and dried overnight. Imaging was performed on an Axiovision upright microscope using a 100x oil-immersion objective and images were captured using an Axiocam color CCD camera and Axiovision software. Images were analyzed for rough morphological features such as cell and nucleus size/shape and granularity. For viability assays, the number of viable cells was counted every other day using Trypan blue and a hemocytometer. For CFU assays, cells were prepared and cultured as per the manufacturer’s protocol (StemCell Technologies, Methocult kit H4435 Enriched). Five individual participant samples were used, two plates per patient sample, and 3x10^5^ cells per plate. Colonies were assessed and scored after 15 days.

### Hoxa3-conditioned medium preparation

HEK293T cells (American Type Culture Collection) were maintained in DMEM supplemented with 10% FBS and 1% P/S. At 20% confluency, cells were transfected with 10 μg SP-Hoxa3-mCherry or SP-mCherry expression plasmids using the calcium phosphate method. Live imaging was used to monitor transfection efficiency (typically 80%). Conditioned medium from transfected cells was collected at 24, 48, and 72 h post-transfection, filtered via a 0.45-μm filter, and used to supplement CD11b^+^ PBMC culture medium at a 1:1 volume for morphological analyses, viability assays, and CFU assays.

### Insect cell-baculovirus transfection and protein purification

For macrophage experiments, purified 6X-His-tagged Hoxa3 or mCherry protein was used. Protein production and purification was performed at the Protein Core Facility at Manchester Institute of Biotechnology. pTriex-Hoxa3-His plasmid or pTriex-mCherry-His plasmid (control) were co-transfected into Sf9 insect cells and with linearized FlashBac Ultra baculovirus DNA (OET: Oxford Expression Technologies) using Escort IV transfection reagent (Sigma Aldrich). The recombinant virus generated was then used to infect a 50 ml suspension culture of Sf9 cells at 2x10^6^ cells/ml grown in Insect-XPRESS media (Lonza) to produce a high titre virus stock. After 1 week, cells were centrifuged (1500 rpm for 10 minutes) and virus-containing media removed, titred by plaque assay, and stored at 4°C. For scale up infection, typically one litre of High 5 insect cells (Thermo Fisher Scientific) were grown to 2x10^6^ cells/ml in Express 5 media with added 20 mM glutamine (Thermo Fisher Scientific) and infected with recombinant virus at a multiplicity of infection of 5 for 72hrs at 28°C with shaking at 110rpm. For protein purification, each 1 litre virally infected culture was first clarified at 1500rpm for 10 minutes and the cell pellet was either directly snap frozen on liquid nitrogen for long term -80°C storage or immediately resuspended in 30 ml of ice-cold lysis buffer (50 mM sodium phosphate pH 8.0, 0.3M sodium chloride, 10% glycerol, 5 mM imidazole, 1% Triton X-100, with 1:500 protease inhibitor cocktail–EDTA [Sigma Aldrich] and 1:10,000 benzonase [Merck]). The lysate was then clarified by centrifugation at 17,000rpm and soluble lysate bound to zinc resin (ABT) at 4°C for a minimum of 2 hours with mixing. The resin was centrifuged at 2000rpm and then batch washed with buffer containing increasing amounts of imidazole (from 10 mM to 50 mM). Bound protein was eluted in buffer containing 250 mM imidazole then buffer exchanged to lower the imidazole concentration with PD-10 desalting column (GE Healthcare). Protein was concentrated using a vivaspin 10K spin column (Generon).

### Dialysis of purified Hoxa3^His^ or mCherry^His^ protein and transduction of exogenous Hoxa3 into human PBMC-Mφ

Purified recombinant proteins were dialyzed to replace the final elution buffer with RPMI medium. Dialysis was performed using Slide-A-Lyzer mini dialysis device (Thermo Scientific, 88401) following the manufacturer’s protocol. Purified and dialyzed Hoxa3^His^ protein or control mCherry^His^ protein was added to the medium of PBMC-derived macrophages on day 6 of cell culture at a concentration of 50 nM for 48 hours. Hoxa3^His^ protein, but not mCherry^His^, was able to passively translocate into macrophages as previously described [20–22].

### Nuclear protein extraction

Nuclear protein was extracted from PBMC-derived macrophages treated with His-tagged Hoxa3 or mCherry (control) protein, or from untreated cells, for further applications using a nuclear extraction kit (ab113474, Abcam) following the manufacturer’s protocol. Protein concentrations of the nuclear samples were measured using the BCA protein assay kit (7780, Cell Signaling Technology) and the absorbance of samples and standards was read at 562 nm on a plate reader (DYNEX Technologies).

### Western blots

Protein samples, either from column purified whole cell lysate (100 ng) or nuclear extracts (20 **μ**g) described above, respectively, were separated by 10% SDS-PAGE and transferred using Trans-Blot Turbo Transfer Pack (Bio-Rad, 170–4157) in a Trans-Blot Turbo transfer system (Bio-Rad) as per the manufacturer's instructions. The blot was incubated with mouse monoclonal anti-6x-His-tag (1:1000, Abcam, ab18184). Protein was detected using Pierce ECL western blotting substrate (Thermo Scientific) and target bands were detected with the ChemiDoc MP System (Bio-Rad).

### Macrophage activation

PBMC-derived macrophages were classically activated using 100 ng/ml LPS (Sigma Aldrich) and 25 ng/ml recombinant human interferon gamma (INF-ɣ; PeproTech) on day 7 of differentiation or were left non-activated. All classically activated and non-activated cells were maintained in serum free medium with 1% Penicillin/Streptomycin (Sigma-Aldrich) and incubated for 1 or 6 hours prior to RNA extraction or collection of protein or supernatant.

### Human IL-6, TNF and Total NF-κB p65 and NF-κB p65 (pS536) ELISAs

All cytokine analyses were performed using ELISA Ready-Set-Go kits (Invitrogen) following the manufacturer’s instructions using supernatants collected from classically activated or non-activated macrophages. Total and phosphorylated NF-κB p65 proteins were measured using the NF-κB p65 (pS536) + total NF-κB p65 SimpleStep ELISA Kit (Abcam, ab176663) following the manufacturer’s protocol. The absorbance in both assays was read on a spectrophotometer at 450 nm.

### RNA extraction, cDNA synthesis and quantitative real-time (qRT)-PCR

RNA was isolated from cells at the time points indicated in the text using TRIzol reagent (Ambion) and treated using the TURBO-DNA-free kit (Ambion) following the manufacturer’s protocol. One microgram of RNA was used for reverse transcription using the Tetro cDNA synthesis kit (Bioline) in a total volume of 20 μl. The resulting cDNA was diluted 1:4.5 for use as template for Taqman qRT-PCR analysis using the following gene expression assays: CD68 (Hs02836816_g1), TNF (Hs00174128_m1), CCL2 (Hs00234140_m1), IL-6 (Hs00174131_m1), CSF1R/M-CSFR (Hs00911250_m1), RUNX1 (Hs00257856_s1), SPI1/PU-1 (Hs02786711_m1), CSF1/M-CSF (Hs00174164_m1) and CEBPA (Hs00269972_s1). ACTB (Hs99999903_m1) and RPL37A (Hs01102345_m1) were used as reference genes. For SYBR^™^ green (Thermo Fisher) reactions, cDNA was diluted 1:7 and mixed with 10 μM forward and reverse primers using the following primer pairs: CD14: F: 5’- CGCTCCGAGATGCATGTG -3’, R: 5’- AACGACAGATTGAGGGAGTTCAG -3’, CD11b: F: 5’- CAACAAGCAGGTCTAGATGGT- 3’, R: 5’- TGAGCCACACACAGAGCTTGCT -3’[23], CD11c: F: 5’-CCGATTGTTCCATGCCTCAT-3’, R: 5’-AACCCCAATTGCATAGCG-3’ [24]. RPL37A was used as reference gene F: 5’- ATTGAAATCAGCCAGCACGC-5’ and R: 5’- AGGAACCACAGTGCCAGATCC-3’ [25]. Reactions were performed using StepOnePlus (Applied Biosystems) and relative expression of genes in tested samples and control were calculated using the delta-delta Ct method. All analyses were performed using Microsoft Excel 2013.

### RNA sequencing

RNA was isolated from non-activated and TNF-activated (3 hours) day 6 macrophages from 3 healthy volunteers and 3 Type II diabetic patients. RNA sequencing was performed by the University of Manchester Genomic Technologies Core Facility on an Illumina HiSeq 4000 machine. The paired-end RNA-seq reads were quality assessed using FastQC (v 0.11.3), FastQ Screen (v 0.9.2). The data was processed with Trimmomatic (v 0.36). The RNA-seq reads were mapped against the reference human genome hg38 using STAR (version 2.5.3a). Counts per gene were calculated by STAR using annotation from GENCODE 30. Normalisation, principle component analysis and differential gene expression tests were performed in DESeq2 v1.20.0.

### Histone acetyltransferase and histone deacetylase activity assays

Histone acetyltransferase (HAT) activity was measured in nuclear extracts that were prepared using the nuclear extraction kit described above, but without the addition of DTT. The concentration of protein extracts was determined with BCA protein assay kit (Cell Signaling). HAT activity was measured using the Histone Acetyltransferase Activity Assay kit (colorimetric) (Abcam, ab65352) as per the manufacturer’s protocol. The optical density (OD) was measured at 440 nm after 2 hours of incubation and HAT activity was expressed as relative OD_440nm_/μg of total protein. Histone deacetylase (HDAC) enzyme activity was measured in nuclear extracts using the HDAC Assay kit (fluorometric) (Active Motif) following the manufacturer’s instructions. Fluorescence was read on a plate reader with 360 nm extraction wavelength and 460 nm emission wavelength. To determine the specific activity of HDAC enzymes, the HDAC standard concentration (μm) was converted into mass (pmol). The enzymatic activity of HDAC in nuclear samples was calculated by dividing the pmol of product formed by the incubation time (minutes) and the mass of HDAC (milligrams) used to yield the activity (pmol/min/mg). The overall enzymatic activity of specific HDACs in all samples, including the positive control (Hela cell nuclear extract) and HDAC inhibitor (Trichostatin A; TSA), was calculated, based on the standard curve.

### Chromatin immunoprecipitation qPCR assay

One million macrophages from each sample were cross-linked for 5 minutes in 1% formaldehyde, then quenched with Tris-HCl pH7.5 to a final concentration of 125mM. Cells were lysed for 5 minutes in 50mM Tris- HCl pH 8.1, 2mM EDTA pH 8.0, 0.1% NP-40, 10% glycerol, 2mM DTT, 1mM PMSF, supplemented with protease inhibitor cocktail (Roche), pelleted, then sonicated in 50mM Tris-HCl pH 8.1, 10mM EDTA pH 8.0, 1% SDS, 2mM DTT, 1mM PMSF (with protease inhibitor cocktail) for 30 rounds of 30 seconds on high in a BioRupter (BioRad). Average fragment size of 300bp was verified by gel electrophoresis. Samples were diluted 1:5 in ChIP buffer (50mM Tris-HCl pH 8.1, 5mM EDTA pH 8.0, 200mM NaCl, 0.5% NP-40, 1mM PMSF) and incubated with Dynabeads Protein A (Life Technologies) for 1 hour at 4°C with rotation to pre-clear the lysate. 25μL total chromatin was set aside, further diluted, and constituted the input sample. 3μg rabbit anti-H3K27Ac (ab4729) or 3μg rabbit IgG (Millipore) was then added to the ChIP samples, and tubes rotated at 4°C overnight to allow antibody binding. Samples were incubated with Dynabeads Protein A for 30 minutes and rinsed once in wash buffer (20mM Tris-HCl pH 8.1, 2mM EDTA pH 8.0, 500mM NaCl, 0.1% SDS, 1% NP-40, 1mM PMSF). Five further washes in wash buffer were then performed, followed by three washes in each of LiCl buffer (20mM Tris-HCl pH 8.1, 2mM EDTA, 1% NP-40, 0.1% SDS, 500mM LiCl, 1mM PMSF), then TE. Samples were eluted from the beads by incubation with 30μL 2% SDS in TE for 15 minutes at 25°C with shaking at 1400rpm, followed by a further 30μL elution step performed at 65°C. Eluates were pooled and crosslinks reversed overnight by addition of 3μL 5M NaCl and incubation at 65°C. For the input sample, reversal of crosslinking was followed by incubation with 0.5μg proteinase K for 1h at 45°C. All samples were column purified (QIAGEN) and eluted in a final volume of 30μL; ChIP and input samples were processed independently.

Primers used to amplify the human TNF promoter were as follows: forward, 5’-CCC-TCC-CAG-TTC-TAG-TTC-TAT-C-3’, and reverse, 5’-GGG-GAA-AGA-ATC-ATT-CAA-CCA-G-3’. SYBR green qPCR was performed on a StepOnePlus Real Time PCR machine using a standard protocol. Fold enrichment over rabbit IgG control was calculated relative to input for the *TNF* promoter after qPCR.

### Plasmids

The pTriex-3-Neo-Hoxa3-His tagged plasmid was made in the Mace lab, and pTriex-3-Neo-mCherry-His-tagged plasmid was made at the Protein Core Facility at the Manchester Institute of Biotechnology. All plasmid maps and sequences are provided as Supporting Information.

### Statistical analyses

Statistical analyses were performed using Microsoft Excel and data are presented as mean ± SEM unless otherwise noted. Data were assumed to be normally distributed. A student *t*-test was used to evaluate significant differences between 2 groups with *p* ≤ 0.05 denoting statistical significance unless otherwise stated. Benjamini-Hochberg corrections for multiple comparisons were performed using a false discovery rate of 0.1. Comparisons that remained significant are reported as such.

## Results

### Macrophages from diabetic patients show excessive production of IL-6 and TNF inflammatory cytokines

**Fig 1:**
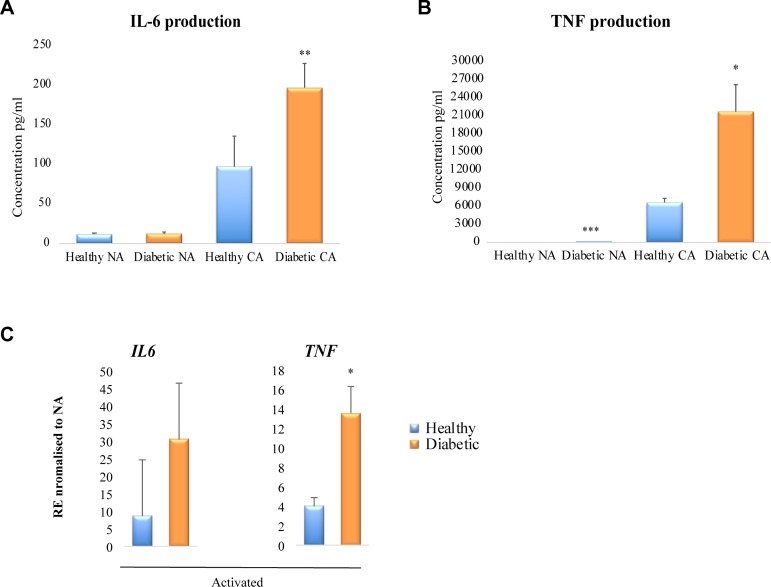
Diabetic-derived human macrophages show increased pro-inflammatory cytokine levels. **(A)** IL-6 cytokine production from ex-vivo derived human macrophages differentiated from PBMCs of healthy volunteers and type II diabetic patients that were either non-activated (NA) or classically activated (CA) by LPS and INF-ɣ. **(B)** TNF cytokine production from ex-vivo derived human macrophages differentiated from PBMCs of healthy volunteers and type II diabetic patients that were either non-activated (NA) or classically activated (CA) by LPS and INF-ɣ. Data represented as pg per ml, mean ± SEM, n = 3 for each NA group, n = 7 for each CA group. **C)** Relative expression (RE) of *IL-6* and *TNF* mRNA in macrophages activated with 10 ng/ml TNF. *p<0.05, **p<0.01, ***p<0.001 in diabetic human patients versus healthy volunteers.

To assess whether diabetes primes human macrophages towards a pro-inflammatory phenotype, as was observed in diabetic mouse macrophages [12–14], the production of IL-6 and TNF inflammatory cytokines was measured from peripheral blood monocyte (PBMC)-derived macrophages from healthy volunteers and diabetic patients. Macrophages were either classically activated with LPS and INF-ɣ, or non-activated. Although there were no differences between non-activated macrophages from healthy volunteers and diabetic patients, IL-6 was significantly increased by 2-fold in classically activated diabetic macrophages compared to macrophages from healthy volunteers (p<0.001; Fig 1A). TNF production was also significantly increased in diabetic-derived macrophages compared to healthy-derived macrophages (3.2-fold increase, p<0.05; Fig 1B). In addition, data from RNA-sequencing of human macrophages showed an increased activation of *TNF* mRNA expression in response to TNF stimulation (3.4-fold increase, p<0.05, Fig 1C). Together, these data suggest that human diabetic macrophages show an excessive production of inflammatory cytokines in response to activation when compared to normal macrophages.

### Hoxa3 protein transduction has no effect on human monocyte morphology or survival

We previously reported that overexpression of Hoxa3 inhibited the pro-inflammatory profile of murine macrophages in wounds in vivo, as well as murine bone marrow-derived macrophages in vitro [19,26]. The importance of this effect of Hoxa3 is underscored by evidence that sustained levels of pro-inflammatory pathways in diabetes plays a major role in creating the chronic wound environment [27–29].

**Fig 2:**
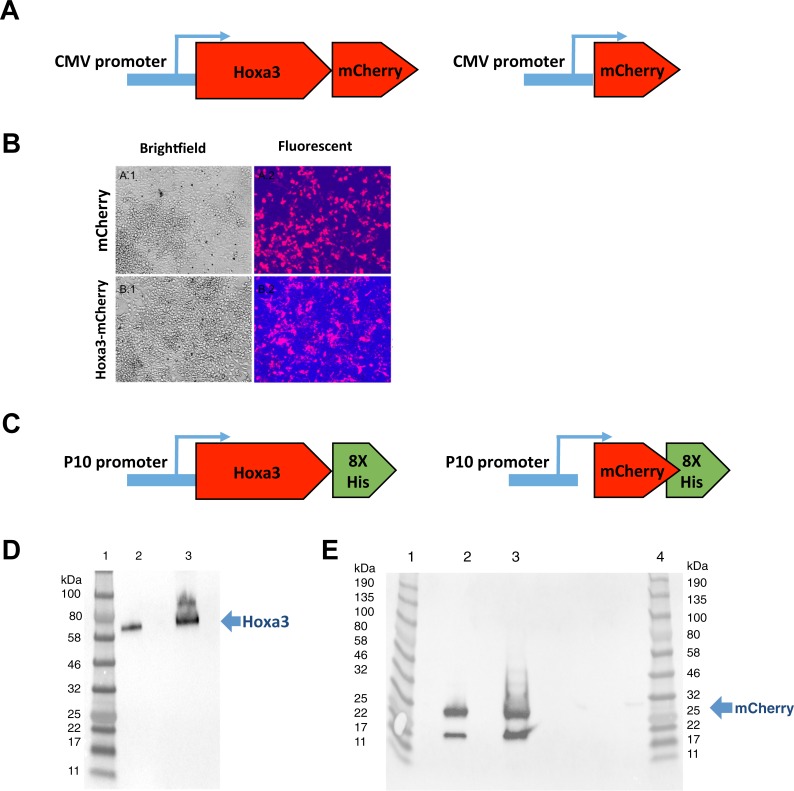
Production of Hoxa3 or mCherry (control) proteins for use in protein transduction. (**A)** schematics of N-terminally tagged sequence peptide (SP)-Hoxa3-mCherry fusion protein and SP-mCherry control constructs driven by the CMV promoter. **(B)** SP-Hoxa3-mCh and mCherry proteins expressed in 293T cells, shown at original magnification x200. **(C)** Schematics of Hoxa3-6X-His and mCherry-6X-His constructs driven by the P10 promoter. **(D)** Purified Hoxa3 protein before and after dialysis analyzed by Western blot using an anti-His-tag antibody. Lane 1: Protein standard, Lane 2: purified Hoxa3-His protein in elution buffer, Lane 3: Hoxa3-His after dialysis with RPMI. **(E)** Purified mCherry-His control protein before and after dialysis analyzed by Western blot using an anti-His-tag antibody. Lane 1: Protein standard, Lane 2: purified mCherry-His protein in elution buffer, Lane 3: mCherry-His after dialysis with RPMI.

**Fig 3:**
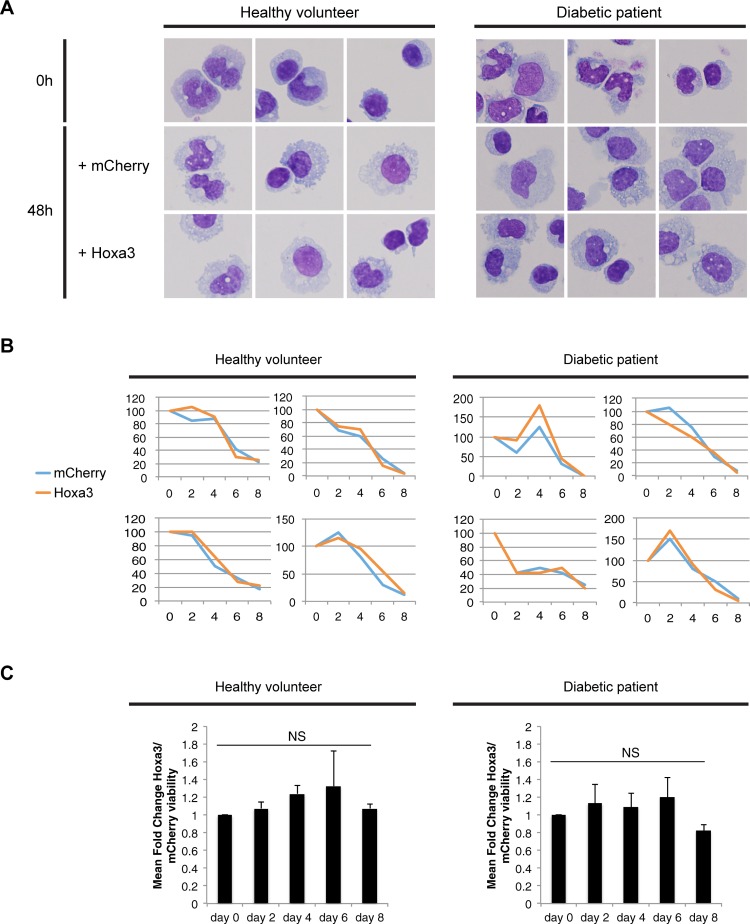
CD11b^+^ PBMC morphology and viability following culture in Hoxa3-mCh or mCh control conditioned media. **(A)** CD11b^+^ PBMC morphology at 0h prior to culture and at 48h following culture in conditioned medium containing either mCherry or Hoxa3-mCh. Cells were cytospun onto microscopy slides and imaged after Giemsa staining (100x magnification). Scale bar = 10 μm. **(B)** The number of viable CD11b^+^ PBMC cells prior to culture with conditioned medium at day 0 and 2, 4, 6 and 8 days post culture in conditioned medium containing either mCherry or Hoxa3-mCh from samples taken from healthy volunteers (left) or from diabetic patients (right), n = 5 for each group. **(C)** Graphs showing means of Hoxa3-treated viable cells in (**B**) normalized to control mCherry-treated. NS = not significant.

The use of protein transduction of Hox proteins was demonstrated to be effective and safe in murine bone marrow cells, and Hoxb4 protein-mediated expansion of human hematopoietic stem cells has been shown to have no leukemogenic effects [30]. However, Hoxa3 protein transduction in human hematopoietic cells has not previously been reported to our knowledge. We therefore aimed to look at the effect of Hoxa3 protein-based treatment on human monocytes and macrophages and whether it had any effects on their morphology or survival. Hoxa3 protein was initially produced using mCherry-tagged constructs (Fig 2A) transfected into HEK293T cells that secreted the proteins (Hoxa3-mCherry and mCherry alone control) into the medium, which was then collected as conditioned medium (Fig 2B, and Al Sadoun et al., 2016). Subsequently, Hoxa3 and mCherry control proteins were produced as purified recombinant His-tagged proteins (Fig 2C) to facilitate protein concentration optimization. Purified recombinant protein was dialysed into DMEM, confirmed by Western blot (Fig 2D and 2E) prior to their addition to macrophages, and used at a concentration of 50 nM for protein transduction of target cells. Human monocytes from healthy volunteers and diabetic patients showed no apparent changes in morphology after 48 hours of Hoxa3 protein transduction compared to control mCherry treatment (Fig 3A). Likewise, survival curves of monocytes cultured for 8 days following protein transduction of Hoxa3 or mCherry showed no significant differences (Fig 3B and 3C).

### Hoxa3 protein transduction has no effect on human monocyte, granulocyte or erythrocyte colony formation

**Fig 4:**
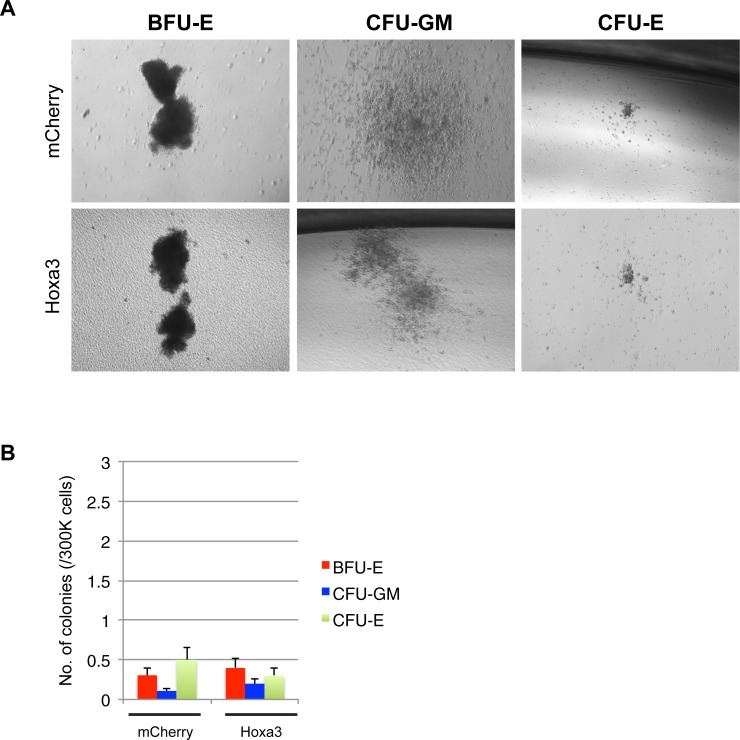
Colony forming assays for CD11b^+^ PBMCs cultured with conditioned medium containing either Hoxa3-mCh or mCherry control protein. **(A)** In vitro colonies of burst forming unit erythroid (BFU-E) (left) or colony forming unit granulocyte-macrophage (CFU-GM) (middle) and colony forming unit erythroid (CFU-E) (right). Colony plates were prepared in duplicate and colonies were analysed at Day 15. Scale bar = 0.5 mm. **(B)** Graphs of colony counts at Day 15 of incubation. Counts show no significant differences between Hoxa3-treated and control-treated cells in the colony forming assays, n = 4 for each group.

CFU assays were performed on CD11b^+^ human monocytes to determine if Hoxa3 protein transduction had any effect on myeloid cell differentiation potential, as myeloid progenitor cell potency is another measure of leukemogenicity. Colonies were counted 15 days after protein transduction with either Hoxa3 or mCherry control. Both treatments showed formation of three colony types: burst-forming unit erythroid (BFU-E), colony-forming unit granulocyte-monocyte (CFU-GM), and colony-forming unit erythroid (CFU-E), with similar frequency (Fig 4A and 4B).

### Exogenous Hoxa3 protein reduces the expression level of pro-inflammatory genes in human macrophages

**Fig 5:**
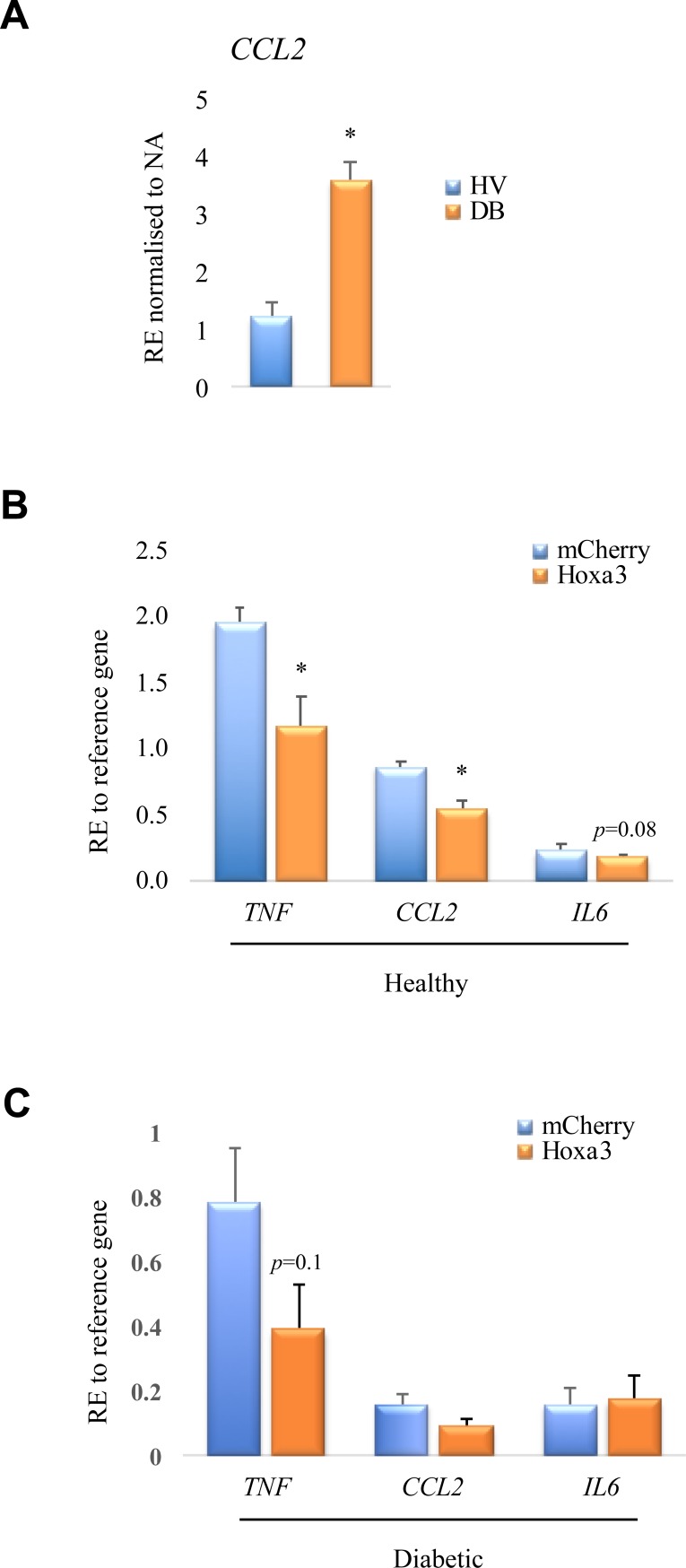
Treatment of human macrophages with Hoxa3 protein suppresses the expression of pro-inflammatory genes. **(A)** Expression of *CCL2* in activated macrophages derived from healthy volunteers and diabetic patients. **(B)** Expression of *IL-6*, *CCL2* and *TNF* pro-inflammatory genes in macrophages derived from healthy volunteers, or **(C)** diabetic patients, treated with Hoxa3 protein or mCherry control classically activated with LPS and INF-ɣ. Data are represented as relative expression to *ACTB* reference gene, mean ± SEM, n = 4 for healthy population, n = 6 for diabetic population, *p<0.05.

Previous studies have shown that another pro-inflammatory gene, chemokine (C-C motif) ligand 2 (*CCL2)*, is overexpressed in macrophages from murine models of diabetes [27–29]. Therefore, we assayed *CCL2* in human macrophages from healthy and diabetic patients and found that *CCL2* is also hyperactivated in response to inflammatory stimulus in diabetic-derived macrophages (p<0.05, Fig 5A). Our previous work has also demonstrated that Hoxa3 overexpression or protein transduction can significantly inhibit pro-inflammatory gene expression [19,26], therefore mRNA expression of *TNF*, *IL6* and *CCL2* was measured in mCherry-control or Hoxa3-treated macrophages from healthy volunteers and diabetic patients, following classical activated. As shown in Fig 5B, Hoxa3-treated healthy-derived macrophages showed significantly reduced expression of inflammatory genes compared to those treated with control, including reduced expression of *CCL2* (p<0.05), *IL6* (p = 0.08) and *TNF* (p<0.05). Hoxa3 treatment showed a similar trend in reduced expression of *TNF* in diabetic-derived macrophages (p = 0.1, Fig 5C) and possibly *CCL2*. These data, therefore, support the hypothesis that treatment of human macrophages with exogenous Hoxa3 protein can attenuate some inflammatory gene expression.

### Hoxa3 protein inhibits IL-6 but not TNF production in human primary macrophages

**Fig 6:**
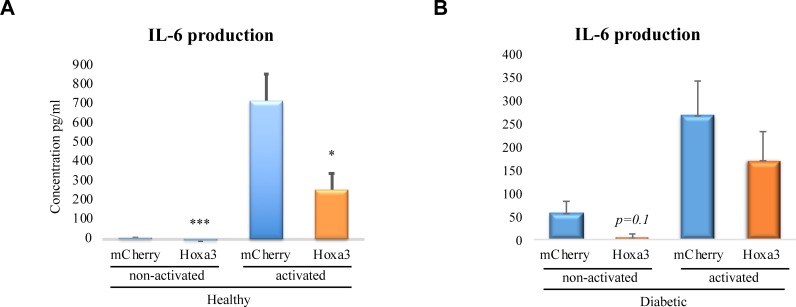
Treatment of human macrophages with exogenous Hoxa3 protein reduces IL-6 production from healthy-derived macrophages. **(A)** IL-6 cytokine production from ex-vivo derived macrophages from healthy volunteers that were treated with Hoxa3 protein versus mCherry control left non-activated (NA) or classically activated (CA) with LPS and INF-ɣ. Data represented as pg per ml, mean ± SEM, n = 3 for each group in NA macrophages, n = 6 for each group in CA macrophages. **(B)**. IL-6 cytokine production from diabetic-derived macrophages treated with Hoxa3 protein versus mCherry control left non-activated (NA) or classically activated (CA) with LPS and INF-ɣ. Data represented as pg per ml, mean ± SEM, (n = 3) for each group in NA, (n = 8) for mCherry-treated CA group, (n = 6) for Hoxa3-treated CA group, *p<0.05, ***p<0.001.

We next tested whether Hoxa3 protein transduction could ameliorate excessive pro-inflammatory cytokine production in human diabetic-derived macrophages as observed in murine cells. Monocyte-derived macrophages from healthy volunteers and diabetic patients were treated with 50 nM of Hoxa3 protein or mCherry control. Cells were left in a naïve state (non-activated) or were classically activated using LPS and INF-ɣ, and production of IL-6 and TNF were then assessed. Although non-activated human macrophages produced minimal amounts of IL-6 in both healthy and diabetic-derived samples, a reduction in IL-6 was evident in response to Hoxa3. Hoxa3-treated cells showed significantly decreased levels of IL-6 from healthy-derived (*p*<0.001; Fig 6A) and a trend in diabetic-derived (*p* = 0.1; Fig 6B) macrophages in unstimulated cells. Following classical activation, IL-6 was markedly upregulated in both healthy and diabetic-derived macrophages treated with mCherry. Treatment with Hoxa3 protein significantly reduced IL-6 production in classically activated macrophages from healthy volunteers by 3-fold (*p*<0.05; Fig 6A). The reduction in IL-6 levels was not significant in Hoxa3-treated classically activated diabetic macrophages (Fig 6B).

**Fig 7:**
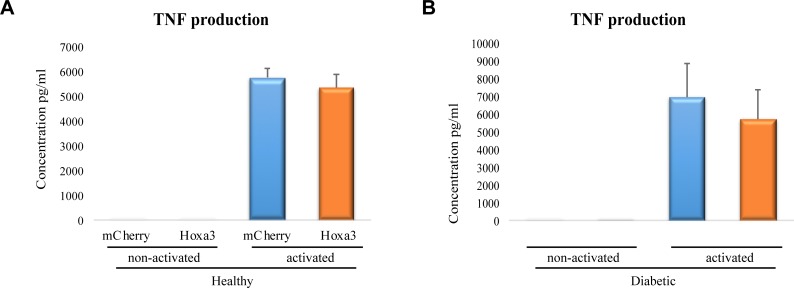
Treatment of human macrophages with Hoxa3 protein does not affect TNF production from healthy or diabetic-derived macrophages. TNF cytokine production from macrophages derived from healthy volunteers **(A),** or diabetic patients **(B),** treated with Hoxa3 protein or mCherry control and non-activated (NA) or classically activated (CA) with LPS and INF-ɣ. Data represented as pg per ml, mean ± SEM, n = 4 for each group in NA and in healthy population, n = 8 for each group in CA diabetic population.

TNF production, however, was not significantly reduced in healthy or diabetic-derived macrophages in response to Hoxa3 protein treatment (Fig 7A and 7B). This discrepancy from mRNA expression could be due to protein stability or high variation in TNF levels among patient samples. Altogether, our data suggest that Hoxa3 protein can limit the production of the pro-inflammatory cytokine IL-6 but not TNF in macrophages in the short term response to pro-inflammatory stimuli such as LPS and INF-ɣ, and may be able to limit TNF production in longer term treatment, since mRNA expression was limited.

### Macrophages isolated from diabetic patients show maturation defects

**Fig 8:**
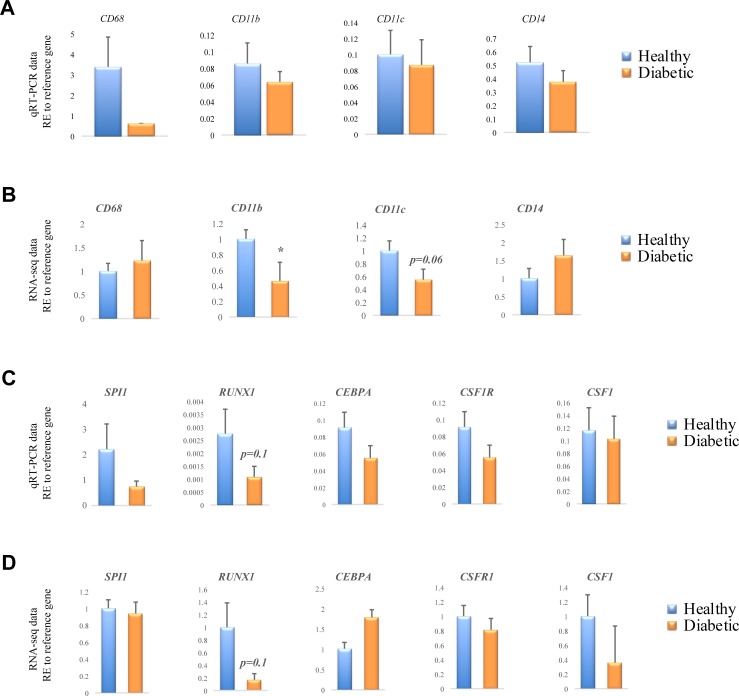
Analysis of macrophage differentiation in diabetic-derived macrophages. Following PBMC isolation, cells differentiated to macrophages using 50 ng/ml of rh-M-CSF for 6 days were assayed for maturation marker expression, **(A)** qRT-PCR analysis of *CD68*, *CD11b*, *CD11c* and *CD14* expression in healthy and diabetic-derived human macrophages (n = 5 for healthy population, n = 7 for diabetic population). **(B)** RNA-seq analysis of *CD68*, *CD11b*, *CD11c* and *CD14* expression in healthy and diabetic-derived human macrophages (n = 3 for each group). **(C)** qRT-PCR analysis of *SPI1*, *CEPBA*, *RUNX1* transcription factors and the *CSF1*-*CSF1R* ligand-receptor pair in healthy and diabetic-derived macrophages (n = 5 for healthy population, n = 7 for diabetic population). **(D)** RNA-seq analysis of *SPI1*, *CEPBA*, *RUNX1* transcription factors and the *CSF1*-*CSF1R* ligand-receptor pair in healthy and diabetic-derived macrophages (n = 3 for each group). Data are represented as relative expression to *RPL37A and ACTB* reference genes, mean ± SEM, *p≤0.05, **p ≤0.01.

We and others reported that macrophages isolated from diabetic mice show maturation defects that begin in the bone marrow and affect their phenotype and function in vivo [12,14]. Immature monocytes/macrophages in mice have been shown to be more pro-inflammatory and are often associated with chronic infection [31]. We therefore tested whether macrophages derived from human diabetic subjects also show maturation defects by assaying macrophage maturation marker gene expression, *CD68*, *CD11b*, *CD11c* and *CD14* in PBMC-derived macrophages from healthy volunteers and diabetic patients. We performed qRT-PCR and RNA-seq and found that mRNA levels of *CD11b* and *CD11c* were consistently reduced and significantly lower in diabetic-derived macrophage RNA-seq data compared to healthy-derived (Fig 8A and 8B). These results suggest that diabetic-derived macrophages have some impairments in maturation compared to healthy-derived macrophages.

We then tested whether diabetic-derived macrophages displayed any differences in the level of master regulator transcription factors that drive myeloid cell differentiation; Spi-1 proto-oncogene (*SPI1*), runt related transcription factor 1 (*RUNX1*), and CCAAT enhancer binding protein alpha (*CEPBA*). These three transcription factors collaborate to directly activate their direct target genes, including the key macrophage differentiation cytokine/receptor pair colony stimulating factor 1 (*CSF1*) and colony stimulating factor 1 receptor (*CSF1R*). We compared qRT-PCR and RNA-seq data and found that the mRNA level of *RUNX1* was consistently lower in macrophages derived from diabetic patients compared to healthy subjects (*p* = 0.1; Fig 8C and 8D). These data demonstrate that diabetes inhibits myeloid cell maturation in human macrophages, similar to findings reported from murine models of diabetes.

### Hoxa3 protein transduction upregulates *RUNX1* and *CD68* in diabetic macrophages

**Fig 9:**
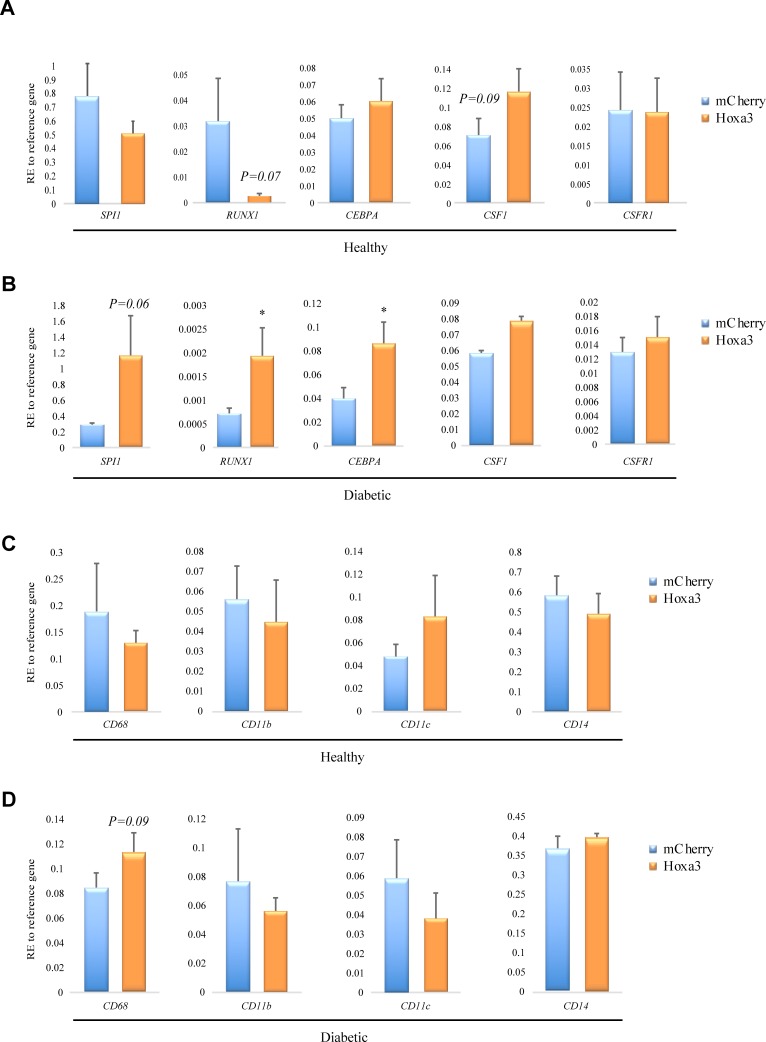
Hoxa3 protein transduction selectively upregulates transcription factors in diabetic-derived macrophages. Expression of *SPI1*, *RUNX1*, *CEBPA*, *CSF1* and *CSF1R*, in Hoxa3 treated vs. mCherry treated human macrophages derived from healthy volunteers **(A),** or from diabetic patients **(B)**. Data represented as relative expression to *ACTB* reference gene, mean ± SEM, n = 4 for healthy population, n = 8 for diabetic population, *p<0.05. Expression of *CD68*, *CD11b*, *CD11c* and *CD14* in Hoxa3 treated vs. mCherry treated human macrophages derived from healthy volunteers **(C),** or diabetic patients **(D).** Data represented as relative expression to *RPL37A* reference gene, mean ± SEM, n = 4 for each population, *p<0.05.

We previously reported that Hoxa3 protein transduction promoted the differentiation of macrophages derived from healthy and diabetic mice [19]. This study also shows that promoting differentiation was an important event prior to inhibiting classical activation. We therefore aimed in the present study to determine whether the attenuation of the inflammatory profile in Hoxa3-treated human macrophages was also associated with promoting maturation potential. Interestingly, we found that in healthy-derived macrophages, Hoxa3 protein transduction significantly downregulated *RUNX1* mRNA (*p*<0.07; Fig 9A), yet upregulated *CSF1* (*p* = 0.09, Fig 9A). In diabetic-derived macrophages, however, Hoxa3 transduction resulted in significant upregulation in the mRNA expression of *SPI1*, *RUNX1* and *CEBPA* (*p*≤0.06, *p*≤0.05, and *p*≤0.05, respectively; Fig 9B), but had no apparent effect on *CSF1* or its receptor *CSF1R*.

We then tested whether Hoxa3 protein treatment could rescue the immature phenotype observed in diabetic human macrophages. Therefore, macrophages derived from healthy volunteers and diabetic patients were cultured for six days and then treated with Hoxa3 protein or mCherry control. The mRNA expression of *CD68*, *CD11b*, *CD11c* and *CD14* maturation markers were subsequently measured. While Hoxa3 protein treatment did not affect the gene expression of healthy human macrophages (Fig 9C), Hoxa3 treatment resulted in significant upregulation of *CD68* in diabetic-derived macrophages compared to control-treated macrophages (*p*<0.09; Fig 9D). Altogether, these data suggest that Hoxa3 protein transduction can promote some aspects of diabetic-derived macrophage differentiation.

### Hoxa3 protein transduction modulates p65/NF-κB and HAT/HDAC activity in activated diabetic macrophages

**Fig 10:**
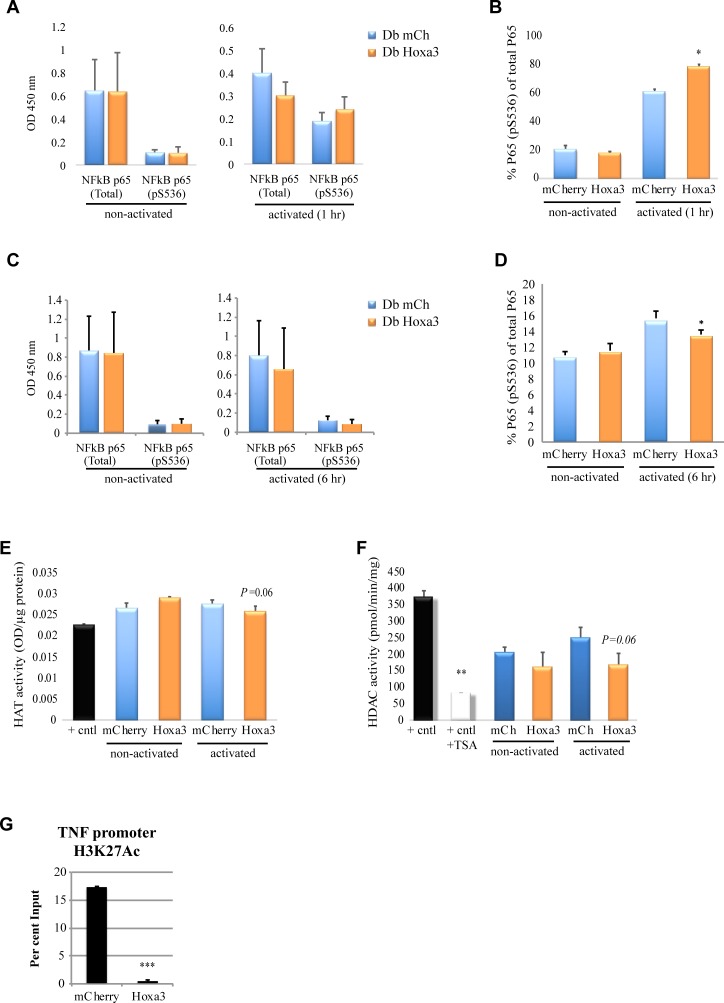
Hoxa3 protein treatment modulates p65/NF-κB and HAT/HDAC activation and inhibits acetylation of the TNF promoter. **(A)** Total and phospho-p65 in Hoxa3-treated vs. mCherry-treated macrophages derived from type II diabetic patients either non-activated (NA) or classically activated (CA) with LPS and IFN-γ for 1 hour. **(B)** Per cent phosphorylated p65/NF-κB (pS536) protein out of total p65 as shown in **(A)**. **(C)** Total and phospho-p65 in Hoxa3-treated vs. mCherry-treated macrophages derived from type II diabetic patients, either non-activated (NA) or classically activated (CA) with LPS and IFN-γ for 6 hours. **(D)** Per cent phosphorylated p65/NF-κB (pS536) protein out of total p65 as shown in **(C)**. Activation of histone acetyl transferases (HAT, OD value per μg of protein), **(E)**, or histone deacetylases (HDAC, pmol/min/mg), **(F)**, analyzed in nuclear protein samples isolated from Hoxa3-treated vs. mCherry-treated macrophages derived from type II diabetic patients either NA or CA with LPS and IFN-γ for 6 hours. HeLa cell nuclear extract was used as a positive control, and Trichostatin A (TSA) used as the control HDAC inhibitor. **(G)** Representative chromatin immunoprecipitation of the TNF promoter (-601 to -230) using anti-H3K27Ac antibody in mCherry- or Hoxa3-treated macrophages from diabetic patients (1 of 4 biological replicates, showing mean + SD of 2 technical replicates, ***p<0.001). All other experiments shown as mean ± SEM, n = 3 for each population, *p<0.05, **p<0.01, ***p<0.001.

We next tested whether Hoxa3 protein treatment could modulate the activity of critical inflammatory mediators p65/NF-κB and HATs/HDACs in diabetic-derived macrophages. Activity of p65 is enhanced by phosphorylation of serine 536 (S536) in the transactivation domain, which results in acute upregulation of p65 target genes. Acute upregulation of inflammatory genes is initially delayed in diabetic myeloid cells upon activation, which may induce prolonged heightened inflammation subsequently upon continuous challenge (Torbica et al., 2019). We found that Hoxa3 enhances early (1 hr) p65 phosphorylation at S536, and thus its activation potential, compared with mCherry-treated macrophages, in classically activated but not in non-activated diabetic macrophages (Fig 10A and 10B). As predicted, longer-term treatment (6 hr) with Hoxa3 resulted in a significant decrease in p65 phosphorylation (Fig 10C and 10D). Moreover, we found that total HAT activity was reduced after 6 hours in diabetic-derived macrophages treated with Hoxa3 protein (*p* = 0.06), compared to mCherry-treated macrophages (Fig 10E). Our results therefore suggest that histone acetylation is inhibited by the effect of Hoxa3 protein.

To investigate whether Hoxa3 protein would also inhibit the activity of class I HDACs in diabetic macrophages, the activity of several HDACs, including HDACs 1, 2, 3, 6, 8, 10 and 11, was assayed. Nuclear extracts collected from diabetic-derived macrophages were treated with Hoxa3 or mCherry protein and were either non-activated or classically activated with LPS/IFN-γ for 6 hours. Reduced activity of HDACs was observed in Hoxa3-treated diabetic-derived macrophages (*p* = 0.06), compared to the mCherry-treated control macrophages under inflammatory conditions (Fig 10F). Finally, as Hoxa3 protein inhibits TNF mRNA expression, and this promoter is known to be regulated in part by acetylation, we analysed the effect of Hoxa3 protein on acetylation of the TNF promoter. All four diabetic patients tested showed a decrease in TNF promoter acetylation in response to Hoxa3 protein transduction. A representative ChIP assay is shown in Fig 10G. Overall, these data indicate that Hoxa3 protein treatment modulates the activity of histone enzymes by reducing the activity of HATs and HDACs, thereby modulating chromatin accessibility at inflammatory loci to signal-induced transcription factors such as p65.

## Discussion

In this report, we demonstrate that PBMC-derived macrophages from diabetic patients show significantly increased levels of the inflammatory cytokines IL-6 and TNF in response to INF-ɣ and LPS stimulation, compared to those from healthy volunteers, supporting the hypothesis that diabetes enhances the pro-inflammatory phenotype of macrophages in human diabetic patients. This result is consistent with other reports showing high levels of inflammatory markers in PBMC-derived monocytes and in fluids extracted from chronic venous ulcers, for instance with a 3-fold increase in TNF levels compared with normal wounds [32]. Overproduction of TNF can impair several processes within the wound including fibroblast and keratinocyte migration, proliferation, apoptosis and production of growth factors [33,34]. Blockade of TNF levels as a therapeutic approach increases the number of proliferating fibroblasts and enhances wound angiogenesis and closure [35,36]. IL-6, on the other hand, is not only accountable for the sustained inflammatory phenotype of the wound, but also the pathogenesis of type II diabetes, as increased IL-6 plays a role in insulin resistance [37].

The results presented in this study suggest that sustained inflammatory responses in macrophages differentiated from diabetic patient PBMCs over 6 days in culture may be the result of aberrant regulation of epigenetic factors, such as chromatin remodelling enzymes, in diabetic myeloid progenitor cells. We have previously shown this in murine models of diabetes; notably, bone marrow (BM)-derived macrophages from *Lep*r^db/db^ mice hyperpolarise in response to LPS and INF-ɣ when cultured in vitro as evidenced by their increased expression of *Tnf*, *Il6*, *Nos2* and *CD86* as well as production of IL-12 and INF-ɣ compared to macrophages derived from non-diabetic littermates [12]. This supports the notion that the dysregulated macrophage phenotype appears in BM-derived cells before they are recruited to the site of injury [12].

Infection and inflammation mediate the release of immature, highly inflammatory hematopoietic cells from the BM to peripheral blood, which is referred to as the left shift [38]. Immature stages of neutrophils and early precursors, such as band and metamyelocytes that normally reside in the BM, are released into the circulation to perform innate immune functions such as phagocytosis, and can resist spontaneous apoptosis in order to eliminate pathogens. In cases of diabetes, a condition associated with chronic inflammation, BM-derived granulocytes and monocytes/macrophages undergo a similar process where they fail to mature fully, exhibiting decreased expression of maturation markers, and showing deregulation of epigenetic factors compared with non-diabetic controls [12,16]. However, investigations of human myeloid cell maturation in diabetes were previously lacking.

We present here the first report to our knowledge that describes maturation defects in macrophages from human diabetic patients, with decreased mRNA expression of maturation markers and transcription factors that drive macrophage differentiation (such as *CD11b*, *CD11c* and *RUNX1*), observed in diabetic-derived macrophages versus healthy-derived controls. Repression of *Cebpa* was previously demonstrated in myeloid cell populations from *Lepr*^*db*/db^ mice and type II diabetic human neutrophils [16]. Here, rescuing the defect in maturation genes via G-CSF-mediated *Cebpa* upregulation in diabetic myeloid cells, rescued the diabetic myeloid cell phenotype and accelerated healing.

The importance of RUNX1 as an early determining factor in myeloid cell fate along with the CEBPA and PU.1/SPI-1 transcription factors has been previously described [39]. However, the roles of these transcription factors are not restricted to maturation as they also promote p65/RelA recruitment to enhancer sites and regulate inflammatory cell activation by LPS [40]. This led us to evaluate whether targeting intrinsic myeloid cell defects in diabetes, with respect to maturational or phenotypic traits, could be rescued in human derived macrophages. Previous work has shown that overexpression of the anti-inflammatory transcription factor Hoxa3 caused an amelioration of the inflammatory phenotype of classically activated BM-derived cells in mice [19]. We therefore evaluated the potential of Hoxa3 to rescue human diabetic myeloid cell behaviour, phenotype and maturation. Here we show that protein transduction of Hoxa3 does not affect macrophage morphology or survival, and does not appear to have any leukemogenic effects as measured by CFU assays. Moreover, Hoxa3 protein transduction inhibited the overproduction of IL-6 in response to LPS and INF-ɣ in diabetic patient-derived macrophages as well as healthy volunteers. Interestingly, although Hoxa3 protein also inhibited TNF promoter acetylation and mRNA expression, it did not significantly alter TNF production from healthy or diabetic-derived macrophages. However, the short versus long-term effects of Hoxa3 treatment were not investigated in this study, and patient-to-patient variation may have contributed to the observed possible effects of Hoxa3 on TNF production. A longer and larger study would focus on distinguishing these possibilities.

At the mRNA level, *CCL2 and IL6* were also downregulated in response to Hoxa3 in healthy-derived macrophages stimulated with LPS and INF-ɣ. However, Hoxa3-mediated inhibition of *IL6* mRNA expression was not found in diabetic-derived macrophages. Again, this discrepancy between mRNA and protein levels indicates further investigation is needed to uncover the mechanism of Hoxa3-mediated regulation of IL-6 protein production.

In terms of macrophage maturation, we report here that Hoxa3 protein transduction rescued the expression of the *RUNX1* myeloid transcription factor. RUNX1, along with CEBPA and SPI-1 synergistically work together to regulate cell type specific genes, myeloid cell differentiation and linage commitment [41,42]. Importantly, in murine macrophages derived from healthy and diabetic mice, we previously found that Hoxa3 upregulated the expression of *Spi1* mRNA and protein levels and that Hoxa3 can physically interact with Spi1 protein and may act as a cofactor in macrophage differentiation [19].

Sustained production of IL-6 and TNF in diabetic macrophages is a consequence of deregulated NF-κB transcription factor function targeting these inflammatory genes [43]. However, chromatin accessibility also plays a critical role in gene regulation [44,45]. RNA-sequencing and bioinformatic analyses of myeloid cells from diabetic mice has identified chromatin regulators as significantly deregulated, with HDAC4 activity specifically shown to be associated with reduced myeloid cell maturation and an aberrant inflammatory phenotype [16]. In this report, we have demonstrated that overexpression of Hoxa3 protein in human diabetic-derived macrophages led to modulation of p65 activity as well as HAT/HDAC activity following activation by LPS and INF-ɣ. This presents a possible mechanism whereby Hoxa3 limits inflammatory gene expression in human macrophages. This is supported by previous reports that demonstrate that inhibition of HDAC activity in fibroblast-like cells and macrophages from patients with rheumatoid arthritis was able to block the production of IL-6 cytokines in those cells [46].

In summary, our results demonstrate that intrinsic defects in macrophage maturation and phenotype are present in human diabetic cells, and appear consistent with the defects seen in diabetic murine macrophages. We also demonstrate that protein transduction with Hoxa3 was a feasible approach to safely reprogram the inflammatory phenotype of human macrophages in terms of their differentiation, sustained inflammation and the chromatin modifications associated with their phenotypic defects. These data reveal the therapeutic potential of Hoxa3 to human macrophages. Future work is required to optimize Hoxa3 protein delivery in vivo in animal models and to human cells, and to gain a deeper understanding of the effects of Hoxa3 on human diabetic macrophages. In particular, further work is needed to understand the mechanisms of Hoxa3 interactions with other myeloid cell transcription factors, such as RUNX1, CEBPA and SPI-1, as well as chromatin modifying enzymes, before translational studies in human clinical trials can be undertaken.

## Supporting information

S1 File datasets This compressed file contains supporting data files for each figure panel. Names of each file correspond to the respective panel.
(ZIP)
